# Hydrogen sulphide-induced hypometabolism in human-sized porcine kidneys

**DOI:** 10.1371/journal.pone.0225152

**Published:** 2019-11-19

**Authors:** Hanno Maassen, Koen D. W. Hendriks, Leonie H. Venema, Rob H. Henning, Sijbrand H. Hofker, Harry van Goor, Henri G. D. Leuvenink, Annemieke M. Coester

**Affiliations:** 1 Department of Surgery, UMCG, University of Groningen, Groningen, the Netherlands; 2 Department of Pathology and Medical Biology, UMCG, University of Groningen, Groningen, the Netherlands; 3 Department of Clinical Pharmacy and Pharmacology, UMCG, University of Groningen, Groningen, the Netherlands; University of PECS Medical School, HUNGARY

## Abstract

**Background:**

Since the start of organ transplantation, hypothermia-forced hypometabolism has been the cornerstone in organ preservation. Cold preservation showed to protect against ischemia, although post-transplant injury still occurs and further improvement in preservation techniques is needed. We hypothesize that hydrogen sulphide can be used as such a new preservation method, by inducing a reversible hypometabolic state in human sized kidneys during normothermic machine perfusion.

**Methods:**

Porcine kidneys were connected to an ex-vivo isolated, oxygen supplemented, normothermic blood perfusion set-up. Experimental kidneys (n = 5) received a 85mg NaHS infusion of 100 ppm and were compared to controls (n = 5). As a reflection of the cellular metabolism, oxygen consumption, mitochondrial activity and tissue ATP levels were measured. Kidney function was assessed by creatinine clearance and fractional excretion of sodium. To rule out potential structural and functional deterioration, kidneys were studied for biochemical markers and histology.

**Results:**

Hydrogen sulphide strongly decreased oxygen consumption by 61%, which was associated with a marked decrease in mitochondrial activity/function, without directly affecting ATP levels. Renal biological markers, renal function and histology did not change after hydrogen sulphide treatment.

**Conclusion:**

In conclusion, we showed that hydrogen sulphide can induce a controllable hypometabolic state in a human sized organ, without damaging the organ itself and could thereby be a promising therapeutic alternative for cold preservation under normothermic conditions in renal transplantation.

## Introduction

Renal transplantation is the preferred treatment for end-stage renal disease[1]. The ongoing increase in the number of renal transplantations and the lack of suitable donors results in an increased use of donation after circulatory death (DCD)[2] and extended criteria donors (ECD). Organs from these donors start with a lower reserve capacity and are more prone to injury caused by warm and cold ischemia, resulting in increased ischemia reperfusion injury (IRI) and graft failure following transplantation[3]. Especially the warm ischemic time, together with extraction- and cooling time, are crucial and relates to survival in renal transplantation[4]. IRI leads, via mitochondrial failure, to cell death, inflammation[5] and fibrosis[6]. In addition, mitochondrial dysfunction might be a surrogate for tissue health after transplantation[7]. Therefore, targeting mitochondria in order to reduce IRI improves preservation of these organs[8]. H_2_S could play a vital role during the process of transplantation[9] and could be a potent therapeutic intervention[10].

Protection against ischemia during the transplantation procedure can be improved by inducing a fast hypometabolic state by directly inactivating mitochondria, instead of the slower cold-forced inactivity. Thereby decreasing the damage obtained by warm ischemia during extraction and bypassing the negative effects of a cold environment. Interestingly, exploiting the gasotransmitter H_2_S to a higher concentration, induces a hypometabolic state in life animals. H_2_S can induce this hypometabolism through reversible inhibition of mitochondrial electron transport chain (ETC), more specifically complex IV (cytochrome c oxidase)[11,12]. Next to inhibition, H_2_S protects the ETC by different mechanisms[13]. Indeed, gaseous administration of H_2_S in mice induces a hypometabolic state of suspended animation[12], prevents renal injury in mice during IRI[14] and is promising in decreasing ROS damage[15–16]. Besides the direct mitochondrial effects[17], H_2_S acts anti-inflammatory[18] and inhibits apoptosis[19]. Although H_2_S showed protective effects during room-temperature static storage[20], until now, neither systemically administered[21] or gaseous administered[22] H_2_S induced successful hypometabolism in larger mammals.

H_2_S is traditionally known for its toxicity with numerous cases of intoxication and death. Though, in these cases of intoxication signs of protection against hypoxic injury are seen supposedly by means of hypometabolism[23]. In the current study, we hypothesize that H_2_S can induce a fast hypometabolic state in isolated perfused porcine kidneys during normothermic machine perfusion at 37 ^o^C and compare it with room temperature at 21°C as measured by oxygen consumption, mitochondrial function and ATP production, without damaging the organ. Possibly being promising new way of organ preservation in donation after cardiac death donors.

## Materials and methods

### Animals

Porcine (female Dutch landrace pigs, 5 months, 130 kilograms on average) kidneys (296 grams on average) were obtained from two abattoirs. Pigs were slaughtered by a standardized legal procedure of a sedative electric shock follow by exsanguination.

### Perfusion

After circulatory arrest, kidneys were exposed to a standardised 30 min of warm ischemia after which they were flushed with 180 ml cold 0.9% saline and connected to a hypothermic perfusion machine (HMP) for 4h, to bridge the time between circulatory arrest and start of the experiment. During HMP the kidnyes were perfused with 500 ml University of Wisconsin solution (UW-MPS, Belzer), 4°C, mean arterial pressure (map) of 25 mmHg, 100 ml/min oxygen supplied.

Before normothermic perfusion, kidneys were flushed with 50 ml cold 0.9% saline to remove the UW-MPS. Afterwards, the kidneys were connected to our normothermic perfusion set-up: map of 80 mmHg, 500 ml of leukocyte depleted blood diluted with 300 ml of Ringer’s lactate and enriched with 7,5 mg/L Mannitol, 7,5 mg/L Dexamethasone, 10 ml 8,4% Sodium bicarbonate, 10 ml glucose 5%, 112,5 mg/L Creatinine, 100mg/200mg Augmentin, 125 ul/L (20mg/ml) sodium nitroprusside, two constant infusion solutions: 82 ml Aminosol, 2.5 ml 8,4% Sodium bicarbonate and 17 IU Insulin (infusion at 20 ml/h) and 5% glucose (infusion at 5 ml/h). The perfusion fluid was oxygenated with carbogen (95% O2 and 5% CO2 at 500 ml/min).

Kidneys were first gradually rewarmed to 21°C during 1h, then warmed to 37°C during 1h, in which the experimental group received 85 mg of the NaHS dissolved in 10 ml 0.9% saline. The infusion of NaHS started after 30 minutes of 37 ^o^C. NaHS was infused at 100 ppm, corrected for the current flow (approximately 5 min). Next, the kidneys were perfused at 21°C for 1h, comparing the possible hypometabolic effect of H_2_S with a temperature drop. 5 kidneys were used per group.

### Perfusion equipment

Perfusion was performed using a Kidney Assist Transporter (Organ Assist, Groningen, the Netherlands) with adjustable software for changing perfusion pressures and a centrifugal pumphead (Deltastream DP3, MEDOS Medizintechnik AG, Germany). Temperature was regulated using a Jubalo water heating system. An integrated heat exchanger (HILITE 1000, MEDOS Medizintechnik AG, Stolberg, Germany) was built in the oxygenator. The flow sensor is a Clamp-on flow sensor (ME7PXL clamp, Transonic Systems Inc., Ithaca, NY, USA). The pressure sensor is a Truewave disposable pressure transducer (Edwards lifesciences, Irvine California, USA).

### Live registration

Oxygen, temperature, flow and diuresis were constantly monitored during the experiment. Oxygen measurements were performed continuously using the PreSens Fibox 4 oxygen-measurement system. Oxygen consumption was shown as (pO_2_ [hPa] arterial–pO_2_ venous[hPa]) * (flow [ml/min] / weight [gr]). Temperature was measured by the integrated sensor of the kidney assist. Flow was constantly measured and noted every 10 min. Urine was constantly collected in a beaker, which was replaced every 15 minutes.

### Biological markers

Serially taken urine and plasma samples were analysed for creatinine, sodium, lactate, pH and potassium at the Clinical Chemical Laboratory of the UMCG. Cortical biopsies were taken for ATP levels (sonification buffer) and histology (formalin). ATP levels were measured using the ATP Bioluminescence Assay Kit CLS II (Roche Diagnostics, Mannheim, Germany) according to standardized protocol and expressed relative to the protein concentration (Pierce^™^ BCA Protein Assay Kit, Rockford, Illinois, USA)[24].

As a marker for reactive oxygen species (ROS) induced damage, lipid oxidation was quantified in tissue samples (taken 90 min after H_2_S infusion) by measurement of malondialdehyde (MDA) using the OxiSelect TBARS assay kit (Cell Biolabs, San Diego, California, USA) according to manufacturer’s protocol, including a butanol extraction. Fluorescence was measured using the Synergy 2 Multi-Mode plate reader (BioTek, Winooski, Vermont, USA). Lipid peroxidation levels were expressed as μM corrected for protein levels (Bradford assay, Biorad, Hercules, California, USA).

### Tissue examination

Periodic acid-Schiff (PAS) staining was performed on the paraffin embedded biopsies taken 75 minutes after H_2_S infusion and analysed by an experienced pathologist.

### Superoxide production with dihydroethidium staining

4 μm kidney biopsy cryosections, taken 90 minutes after H_2_S infusion, were placed on slides and washed three times with phosphate buffered saline (PBS). Thereafter, sections were incubated for 30 minutes at 37°C in darkness with 10μM dihydroethidium (DHE) (Sigma, St. Louis, MO). Sections were washed twice with PBS and scanned with a Leica inverted fluorescence microscope with a 40X magnification. From every kidney coupe, 5 different Images were made to secure a representative area of the total coupe containing all cell types. Every image was scored in a quantitative matter using ImageJ (National Institute of health, Bethesda, Maryland, USA). Proper threshold settings were analyzed using positive control coupes and were found to be between 42 and 255. For every image, the area of fluorescence relative to the total area of the coupe were determined together with the mean fluorescent signal. These two parameters together give an indication of the area and severity of superoxide production.

### Mitochondrial function

Mitochondria were freshly isolated from cortical renal tissue using a standard differential centrifugation protocol and protein concentration was determined (Bradford, Biorad, Hercules, California, USA). 5 ug of mitochondria were resuspended in a total volume of 100 ul mitochondrial buffer containing JC-1 (Sigma Aldrich, Saint Louis, Missouri, USA) with NaHS (0–5 mM) or Carbonyl cyanide-p-trifluoromethoxyphenylhydrazone(FCCP, 2uM). After 30 min incubation (37°C), mitochondrial membrane potential was fluorescently measured by quantifying the fluorescence emission shift from green (529 nm) monomers to red (590 nm) aggregates. Data expressed as ratio red / green, relative to control.

### Statistics

Data were analysed using SPSS 25.0 (SPSS inc., Chicago, IL, USA). An area under the curve analysis was performed in Graphpad PRISM, followed by Mann-Whitney U in SPSS. Analysis was done on the data during H_2_S treatment for O_2_, ATP and diuresis and during and after H_2_S treatment for FE_na,_ creatinine clearance, LDH and ASAT. Kruskal-Wallis H Test was used to analyse the lipid peroxidation. Graphpad PRISM 5.04 (GraphPad, San Diego, CA, USA) was used to create the graphs.

## Results

### H_2_S infusion induces a rapid and reversible decrease in oxygen consumption

**Fig 1:**
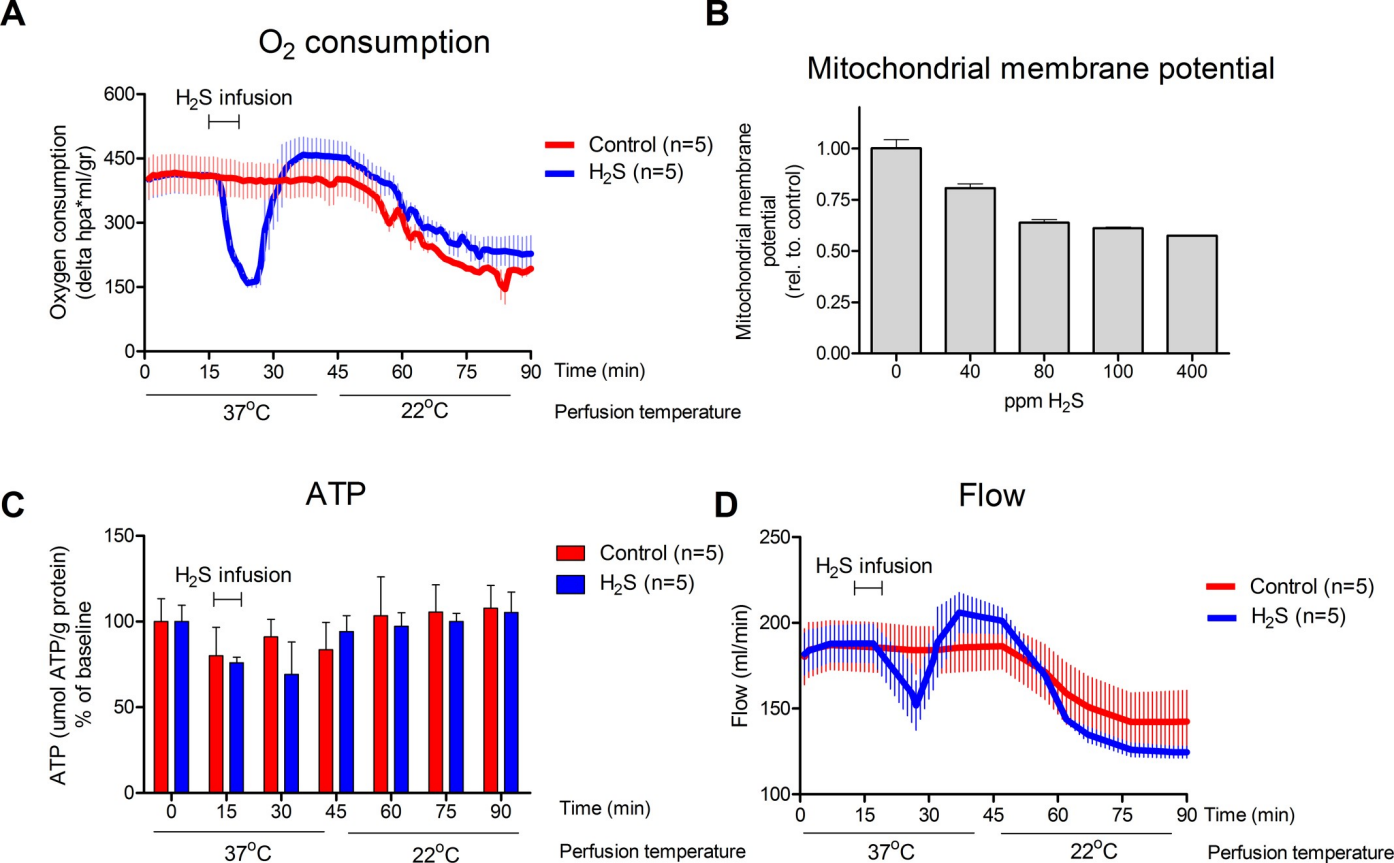
H_2_S effects on kidney perfusion and oxygen consumption. **A.** After H_2_S infusion at 37 ^o^C, a significant (p = 0.047) decrease from 409 ΔhPa⋅ml/min/gr to 160 ΔhPa⋅ml/min/gr is seen which restores to normal oxygen consumption levels with a temporary increase within 20 minutes after NaHS infusion. **B.** Mitochondrial membrane potential in H_2_S treated pig kidney mitochondria (data expressed as ratio red / green relative to control) show a 39% decrease in mitochondrial membrane potential in 100 ppm NaHS treated mitochondria compared to non-treated mitochondria. **C.** ATP levels in renal tissue (data expressed as μmol ATP/g protein as of baseline) show no clear alteration after H_2_S infusion but remain higher after infusion of H_2_S. **D.** As a result of H_2_S administration, flow reduced from 188 ml/min till 152 ml/min. After 20 minutes, the reduced flow restored to slightly above normal levels at 206 ml/min but restores to control levels within 40 minutes after NaHS administration. Figure A, B, D, presented as mean + SEM.

Immediately upon H_2_S infusion, oxygen consumption significantly (p = 0.047) decreased strongly from 409 to 160 ΔhPa⋅ml/min/gr (Fig 1A), but restored rapidly after ending the H_2_S administration. Interestingly, a temporary increase of 20 min in oxygen consumption was observed after the H_2_S infusion, which is not significantly different from the control group, p = 0.602. To compare the hypometabolic effects of H_2_S to hypothermia, we cooled the organ at the end of the experiment. Gradually cooling the kidney to 21°C decreased oxygen consumption to 220 ΔhPa⋅ml/min/gr (Fig 1A).

To examine whether the drop in oxygen consumption is a result of mitochondrial depression, mitochondrial membrane potential was measured in H_2_S treated mitochondria. Increasing NaHS concentrations resulted in decreased mitochondrial activity, were 100 ppm NaHS resulted in a strong decrease in mitochondrial membrane potential compared to non-treated mitochondria (Fig 1B). Despite the decrease in mitochondrial activity during H_2_S infusion, ATP levels did not alter directly after H_2_S infusion between 15 and 45 minutes, P = 0.465 (Fig 1C).

Besides the inhibited metabolism, H_2_S decreased the flow shortly during H_2_S infusion, after which an increase can be seen (Fig 1D). In addition, during cooling the flow decreases.

### Preserving effects of H_2_S on renal function

**Fig 2:**
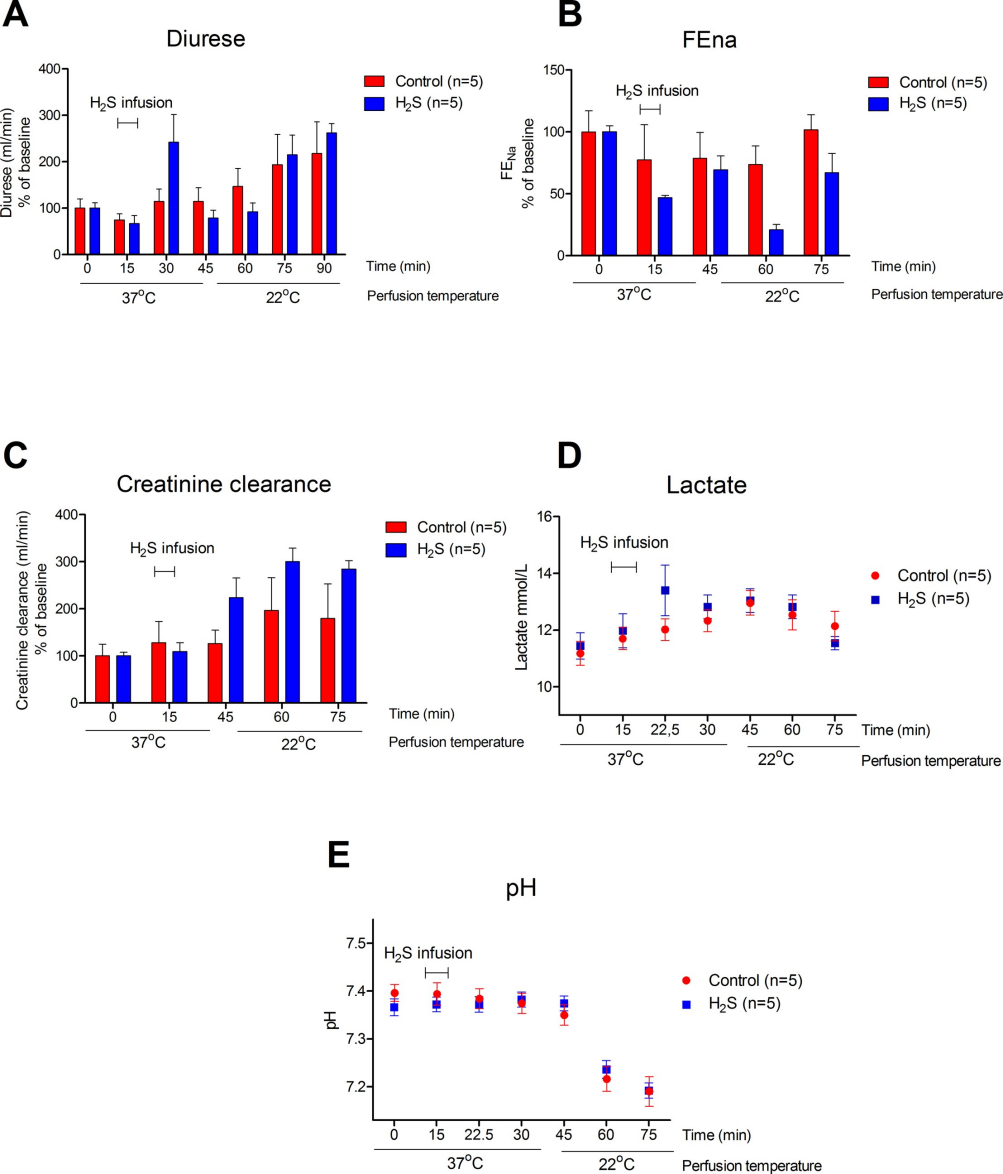
Kidney function during H_2_S treatment. **A.** Diuresis (mL) showing a rigorous increase after H_2_S infusion and restored to control levels within 30 minutes. Data expressed as % of baseline **B.** Fractional excretion of sodium (FEna) showing difference between the H_2_S and control group. Data expressed as % of baseline **C.** Creatinine clearance (mL/min) showing no difference between the H_2_S and control group. Data expressed as % of baseline. **D.** lactate level (mmol/L) of perfusion fluid showing a higher venous lactate level of the H_2_S treated group after infusion of H_2_S. **E.** pH level of perfusion fluid.

During H_2_S infusion diuresis was increased more than double, which restored to control levels within 30 min (Fig 2A, p = 0.175). Renal function, expressed as Fractional excretion of sodium (FEna) shows a non-significant effect on function (Fig 2B, p = 0.465) in the H_2_S treated group, whereas creatinine clearance, show a trend (Fig 2C, p = 0.175) upon H_2_S treatment. Comparable venous lactate levels were seen (Fig 2D), without alterations in pH (Fig 2E).

### No damage response was observed after the H_2_S treatment

**Fig 3:**
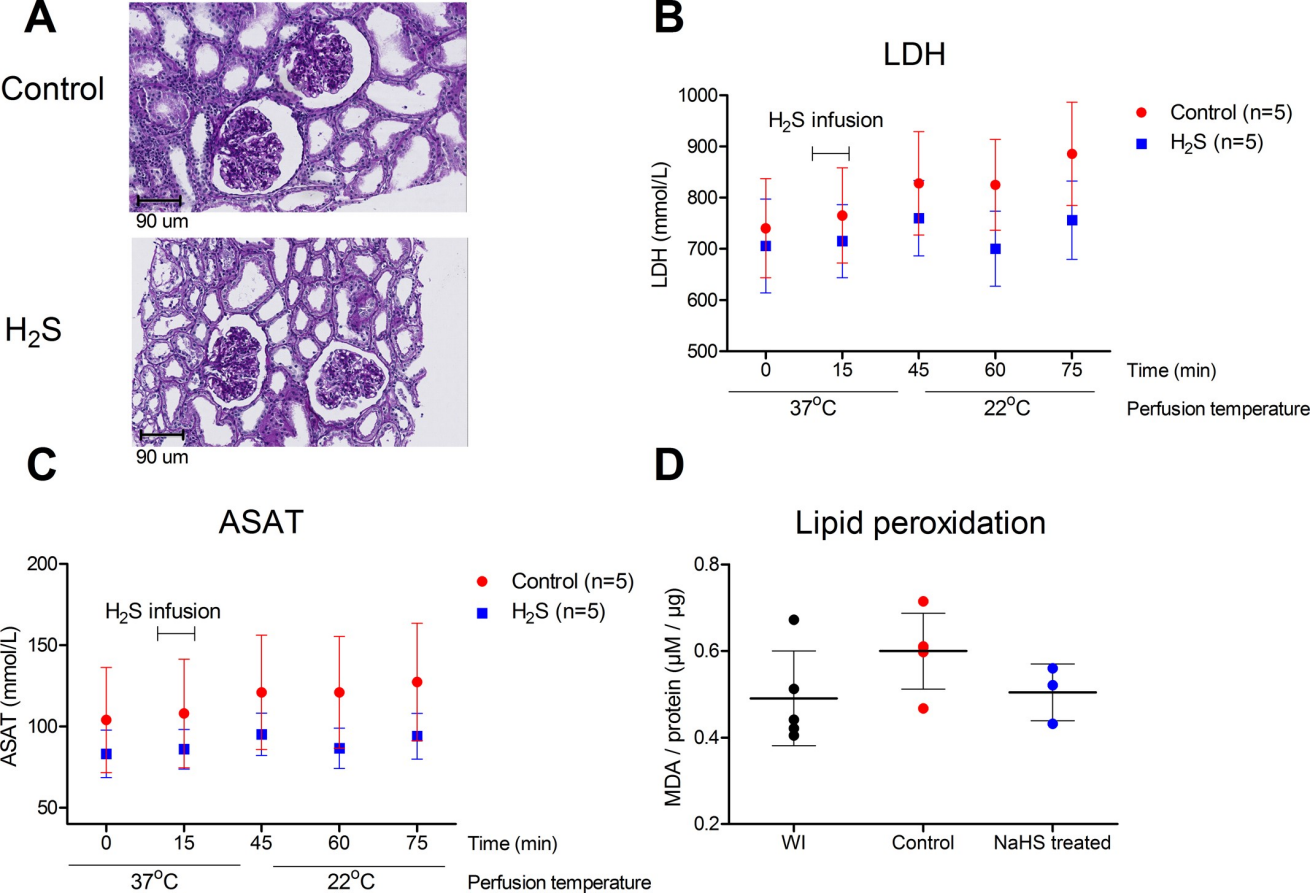
Renal damage response. **A.** PAS stained tissue with comparable histological appearance between H_2_S treated and control. **B.** LDH levels in the perfusion fluid (mmol/L) showed no difference between the H_2_S treated and control group. **C.** ASAT levels in the perfusion fluid (mmol/L) showed no difference between the H_2_S treated and control group. **D.** Lipid peroxidation, expressed as μM corrected for protein level, showed a trend towards decreased MDA levels in the H_2_S treated kidneys. Data expressed as mean with SEM.

**Fig 4:**
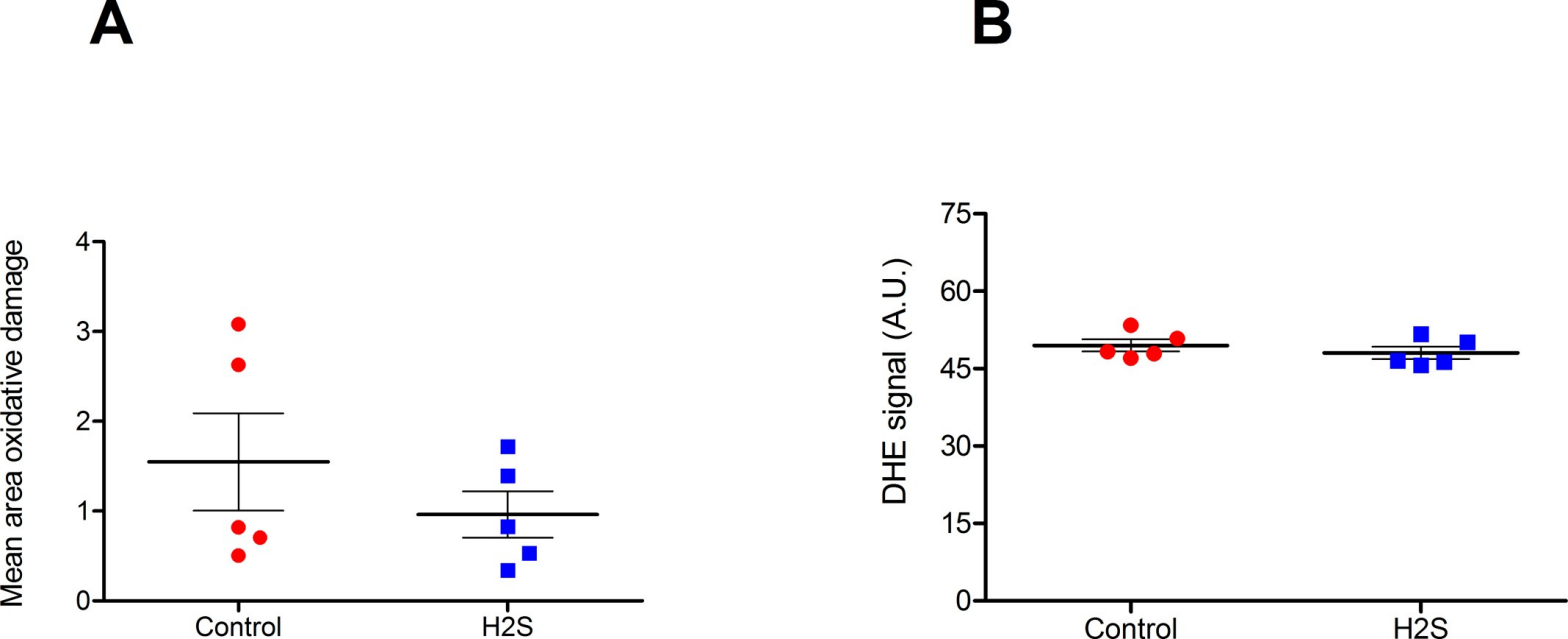
Renal superoxide production measured with dihydroethidium (DHE) fluorescence in control and H_2_S perfused porcine kidneys. **A.** Mean area in which superoxide damage was found, **B.** Mean fluorescent signal. Data expressed as mean with SEM. 40X magnification.

Kidneys were histologically examined for tubular necrosis and ischemic damage, showing comparable histologically appearance between H_2_S and control kidneys (Fig 3A). ASAT and LDH showed a small increase over time, but no differences were observed between the H_2_S treated and non-treated groups (p = 0.602 and p = 0.917) (Fig 3B and 3C). As a marker for reactive oxygen species (ROS), lipid peroxidation was measured in samples before and after perfusion with H_2_S. The H_2_S-induced hypometabolic state did not lead to increased oxidative damage. On top of that, we found a trend of protection substantiated by decreased MDA levels in the H_2_S treated kidneys (p = 0.154) (Fig 3D). Moreover, in DHE stained slices the H_2_S group shows a non-significant trend towards lower superoxide production (Fig 4).

## Discussion

Traditionally, kidneys are preserved by cold storage or, more recently, by hypothermic machine perfusion. Both approaches are based on cold temperatures, lowering the metabolism and prolonging safe conservation of the organ compared to warm ischemia[25]. However, hypothermia is known to be detrimental to cellular processes[26]. Recently we reported that lowering temperatures results in a progressive discrepancy between lowering of mitochondrial respiration and their production of ROS[27]. Furthermore, the length of cold ischemic times is related to an increased risk of graft failure and/or mortality following renal transplantation[28]. This indicates that improved preservation techniques are needed.

We showed that H_2_S can induce a safe and reversible hypometabolic state in human sized porcine kidneys during isolated normothermic perfusion, as shown by decreased oxygen consumption and mitochondrial activity without any short-term damage and signs of renal function improvement. Therefore, H_2_S proved to be a very promising alternative protective method. By inducing a hypometabolic state, H_2_S reduces ischemic injury[14] by scavenging ROS[15–16] and inhibiting apoptosis[19,29]. H_2_S treatment can mitigate renal graft IRI during cold storage following rat-renal transplantation[30] and shows potentially cytoprotective and antiinflammatory effects following renal IRI in CLAWN miniature swine[31]. The hypometabolic effect of H_2_S combined with (sub)normothermic preservation and human sized organs is still unknown.

H_2_S is a gasotransmitter, produced by the conversion of L-cysteine by cystathionine β-synthase (CBS), cystathionine γ-lyase (CSE) and cysteine aminotransferase (CAT), all three mainly located in the cytosol. Additionally, H_2_S is produced directly within mitochondria by 3-mercaptopyruvate sulfur-transferase (3MST)[32]. CBS and CSE translocate to mitochondria during cellular stress such as hypoxia[33]. Displaying the considerable role of mitochondria in H_2_S production and regulation.

H_2_S suppresses metabolism via reversible inhibition of mitochondrial complex IV (also known as cytochrome c oxidase)[34]. This mechanism has been proposed as the driven force behind the hypometabolic state induced by H_2_S when used in high dosages, as in our experiment. A shift towards increased glycolysis could be expected due to loss of the mitochondrial energy production by decreased oxidative phosphorylation. Though, no increase in venous lactate levels or pH was found. Interestingly, ATP levels did not alter after H_2_S infusion, and were comparable to controls after the infusion.

The fast but limited effects of H_2_S on different parameters, such as ATP, can be explained by the H_2_S concentration and time of infusion. NaHS, as very rapid acting H_2_S donor, is known to increase the H_2_S concentration fast, after which H_2_S is rapidly lost from the solution by volatilization in laboratory conditions or transferred across respiratory membranes[35], in this experiment, the oxygenator. This might explain the short and limited effects of H_2_S on injury markers. In addition, the moment of infusion, halfway normothermic perfusion, limited the potential protective properties[14]. Moreover, we used 100 ppm of H_2_S during the experiments, where lower levels could provide a protective effect as well[36]. Since quantification of levels of H_2_S was not performed, the actual amount of H_2_S in the system is unknown.

We showed a complete restoration to normal kidney function after H_2_S treatment. Biochemical parameters (ASAT and LDH) were not altered by H_2_S treatment and histology showed no difference, indicating that short-term damage is absent. In addition, as mitochondrial ROS production is one of the major damaging routes during IRI[37], and H_2_S is a known for ROS inhibition[36], we evaluated lipid peroxidation levels as a marker for ROS before and after perfusion. Although a trend of decreased MDA was seen in the experimental group together with a trend of lower superoxide production in the DHE stained slices, no significant differences were found suggesting that a longer suppression of metabolism with H_2_S could be even more beneficial.

The effect of H_2_S on the increase of diuresis and flow can be explained by vasorelaxation, as seen in earlier experiments in rats[38]. Vasorelaxation and decreased blood pressure, caused by opening of K_atp_ channels[38], can both influence the flow and diuresis. Moreover, similar effects of decreased blood pressure have been seen in a porcine reperfusion model[16]. In addition, CSE knockout mice develop hypertension, indicating that endogenously produced H_2_S modulated blood pressure[39]. The absence of changes in creatinine clearance an FEna advocates that H_2_S does not affect renal function. When H_2_S substrate l-cysteine is infused into renal arteries of rats it causes an increase in GFR and urinary excretion of Na+ and K+[40], explaining a possible better renal function when H_2_S is infused for a longer period of time.

Changing the temperature at the end of the experiment gave insight in the temperature dependent metabolic rate of the kidney and provided an internal control. Cooling is associated with a lower metabolic rate[27]. Indeed, we found a decrease in oxygen consumption when lowering temperature. A comparable decrease in oxygen consumption was found in our H_2_S experiment. Attributing to the decreased oxygen consumption, a lowered MMP was found in both cooled human epithelial kidney cells (HEK293)[27] and H_2_S treated isolated mitochondria, showing similar effects between temperature and H_2_S on the metabolic rate. However, whereas cooling is known for negative effects on the ROS scavenging activity[27], H_2_S is a known scavenger, favouring the use of H_2_S instead of hypothermia.

To summarize, our model shows that a reversible hypometabolism can be induced using H_2_S in human-sized organs. H_2_S could be used clinically at different moments during renal donation and transplantation procedures. Instead of waiting until the organ has cooled during extraction of the organ, inducing a fast hypometabolic state by infusion of H_2_S could reduce ischemic injury. In addition, H_2_S can be used during transportation of the organ, thereby inducing a hypometabolic state in normothermic circumstances, potentially avoiding the deleterious effects of low temperatures. In addition, its antioxidant capacity could reduce IRI[15].

This study shows that H_2_S is applicable for clinical purposes by means of its capacity to induce a rapid reversible state of hypometabolism in the absence of functional or structural deterioration. More research is needed to determine long term effects of H_2_S and its use in the transplantation setting.

## Supporting information

S1 Dataset Dataset of Figs 1–3. (PZF)

S2 Dataset Dataset of Fig 4. (XLSX)
